# Socioeconomic status and social relationships in persons with spinal cord injury from 22 countries: Does the countries’ socioeconomic development moderate associations?

**DOI:** 10.1371/journal.pone.0255448

**Published:** 2021-08-13

**Authors:** Christine Fekete, Jan D. Reinhardt, Mohit Arora, Julia Patrick Engkasan, Mirja Gross-Hemmi, Athanasios Kyriakides, Marc Le Fort, Hannah Tough

**Affiliations:** 1 Swiss Paraplegic Research, Nottwil, Switzerland; 2 Department of Health Sciences and Medicine, University of Lucerne, Lucerne, Switzerland; 3 Institute for Disaster Management and Reconstruction of Sichuan University and Hong Kong Polytechnic University, Sichuan University, Chengdu, China; 4 John Walsh Centre for Rehabilitation Research, Kolling Institute of Medical Research, Royal North Shore Hospital, St Leonards, Australia; 5 Sydney Medical School - Northern, Faculty of Medicine and Health, The University of Sydney, Sydney, Australia; 6 Department of Rehabilitation Medicine, University of Malaya, Kuala Lumpur, Malaysia; 7 Spinal Cord Injuries Unit, University of Patras, Patras, Greece; 8 Neurological Physical and Rehabilitation Medicine Department, University Hospital, Nantes Cedex, France; Bangalore Baptist Hospital, INDIA

## Abstract

**Background:**

Social relationships are powerful determinants of health and inequalities in social relationships across socioeconomic status (SES) groups may contribute to social inequalities in health. This study investigates inequalities in social relationships in an international sample of persons with spinal cord injury and explores whether social gradients in relationships are moderated by the countries’ socioeconomic development (SED).

**Methods:**

Data from 12,330 participants of the International SCI Community Survey (InSCI) performed in 22 countries were used. We regressed social relationships (belongingness, relationship satisfaction, social interactions) on individual SES (education, income, employment, financial hardship, subjective status) and countries’ SED (Human Development Index) using multi-level models (main effects). To test potential moderation of the SED, interaction terms between individual SES and countries’ SED were entered into multi-level models.

**Results:**

Paid work, absence of financial hardship and higher subjective status were related to higher belongingness (OR, 95% CI: 1.50, 1.34–1.67; 1.76, 1.53–2.03; 1.16, 1.12–1.19, respectively), higher relationship satisfaction (OR, 95% CI: 1.28, 1.15–1.42; 1.97, 1.72–2.27; 1.20, 1.17–1.24, respectively) and fewer problems with social interactions (Coeff, 95% CI: 0.96, 0.82–1.10; 1.93, 1.74–2.12; 0.26, 0.22–0.29, respectively), whereas associations with education and income were less consistent. Main effects for countries’ SED showed that persons from lower SED countries reported somewhat higher relationship satisfaction (OR, 95% CI: 0.97, 0.94–0.99) and less problems with social interactions (Coeff, 95% CI: -0.04, -0.09- -0.003). Results from moderation analysis revealed that having paid work was more important for relationships in lower SED countries, while education and subjective status were more important for relationships in higher SED countries (interaction terms *p*<*0*.*05*).

**Conclusion:**

Social relationships in persons with spinal cord injury are patterned according to individual SES and the countries’ SED and larger socioeconomic structures partly moderate associations between individual SES and social relationships.

## Introduction

Social relationships are powerful determinants of health [1–5] and inequalities in accessing and maintaining good quality relationships across groups with different socioeconomic status (SES) may contribute to social inequalities in health [1–3, 6–9]. Persons with lower SES are engaged in smaller and less diverse social networks [10–13], report lower contact frequency [14], receive less social support [6, 7, 11, 15–17], are less satisfied with their social support [14], and more often experience social isolation and loneliness [18–21]. Berkman and Krishna’s conceptual model of how social networks affect health illustrates the importance of structural conditions for social relationships, suggesting that social structures directly influence the extent and shape of people’s social networks in terms of size, density and contact frequency [2]. Social networks subsequently influence opportunities to benefit from psychosocial resources provided by social relationships (e.g. support, feelings of belongingness), which ultimately affect health through behavioral, psychosocial and physiological pathways [2]. Theories of homophily and social capital offer additional insights into mechanisms leading to social inequalities in relationships. Homophily describes the tendency that relationships are commonly formed and maintained between individuals sharing similar characteristics, such as comparable SES [22–24]. Social capital theories maintain that persons with higher SES are more likely to accumulate social capital (i.e., access to and use of resources embedded in social networks) than persons with lower SES [25], as they are seen as attractive partners for social exchange processes [22]. Social inequalities in the access to and the maintenance of health-promoting social relationships thus result from unequal access to social capital and the tendency towards clustering of individuals with similar SES in social networks (homophily), leading to an aggregation of health-related resources provided by social capital among higher status groups that tends to be not relayed to lower status groups.

Social relationships may not only differ by individual SES, but also according to larger structural conditions and cultural norms, such as a countries’ socioeconomic development (SED), the population’s education level, and cultural habits on living arrangements and family dynamics [2, 26]. Macro-level structural conditions, such as social cohesion, competition, inequality, discrimination, and poverty, influence social networks and ultimately functional aspects of social relationships and countries with poorer structural conditions may therefore provide less favorable circumstances for the development of functional social relationships [2, 27, 28]. These assumptions are empirically supported by a recent report from the European Commission stating that around 10% of persons in Hungary, the Czech Republic, Italy, Poland, France and Greece frequently feel lonely, while at most 5% of persons from the Netherlands, Denmark, Finland, Germany, Ireland and Sweden regularly feel lonely [29]. This pattern may relate to the countries’ SED, as mostly people from countries with higher SED, such as Scandinavian countries and Germany reported lower loneliness levels than people from countries with lower SED, such as Eastern and Southern European countries. Similarly, analysis of pooled data from waves 1–8 of the European Social Survey showed that 10% of people from Scandinavian countries reported few social contacts (never to once a month), with a dramatically higher share of 31.8% in Eastern European countries [30].

Besides main effects of individual SES and the countries’ SED on social relationships, it is also likely that the countries’ SED *moderates* associations between individual SES and social relationships. A moderator is understood as a third variable that increases or decreases the strength and/or direction of the association between predictor and outcome and moderation occurs if the predictor-outcome association differs depending on the value of the moderator [31]. Cross-level interaction effects are defined as interactions in clustered data in which the moderator is a higher-level variable [32, 33], such as the countries’ SED. A cross-level interaction effect in the context of social inequalities in relationships would indicate that associations between individual SES and social relationships depends on the countries’ SED. We would expect that low individual SES has more detrimental effects on social relationships if larger structural conditions are unfavorable, such as in countries with lower SED.

Social relationships might present an even more critical resource in persons with disabilities as they are often instrumental facilitators to overcome various disability-related barriers [34, 35]. Although the importance of social relationships for health in people with physical disabilities has received considerable attention in research [36, 37], studies on social inequalities in relationships in persons with disabilities are scarce [38], and studies taking into account structural conditions, such as the countries’ SED, are currently unavailable in disability research. The countries’ SED may directly affect social relationships of persons with disabilities as environmental barriers for social participation including discrimination against and misconceptions about persons with disabilities [39–41] differ according to a countries’ SED [42, 43].

**Fig 1:**
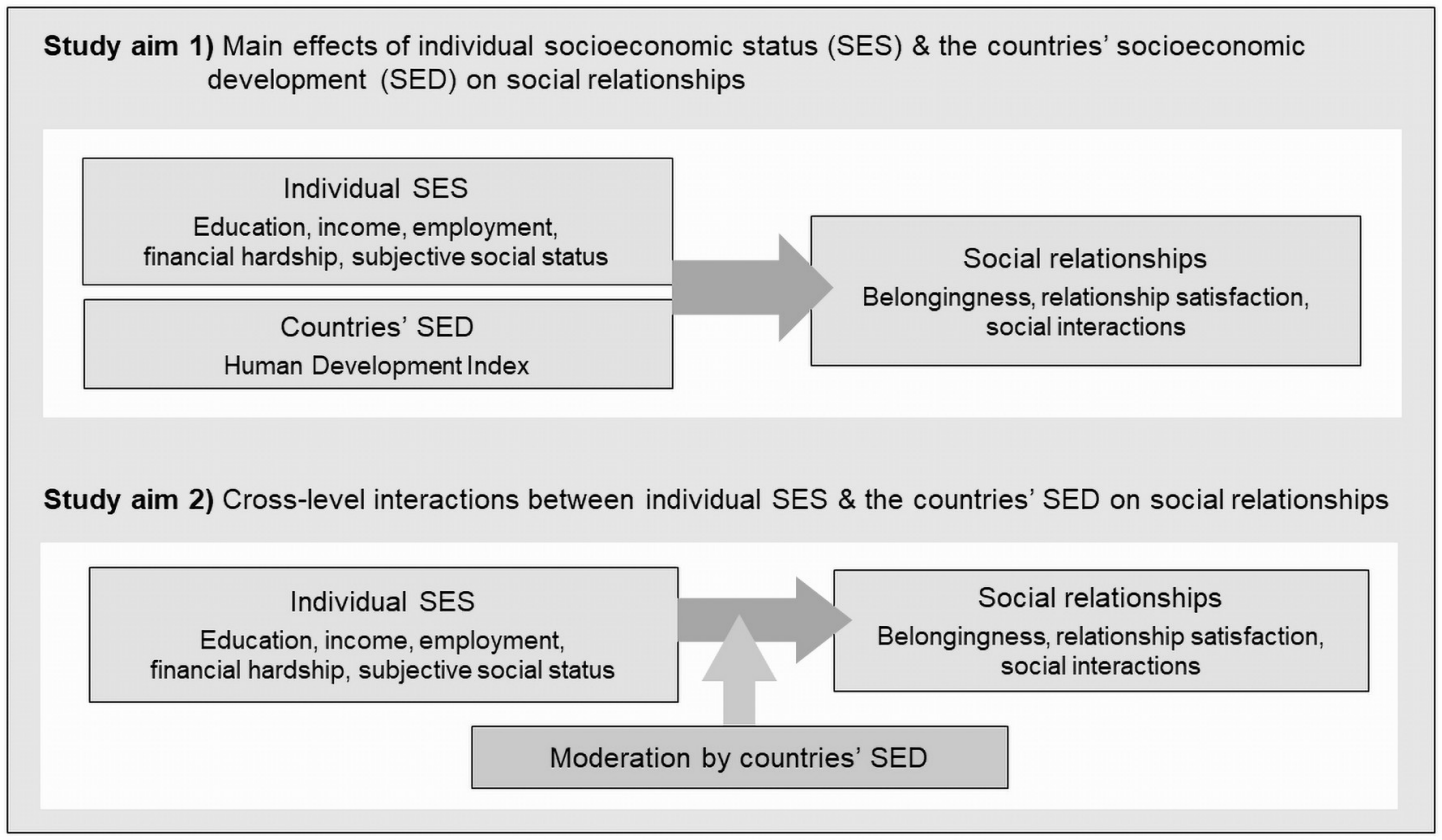
Analytical framework of the study.

Although social relationships provide important resources to overcome disability-related environmental barriers and maintain health of persons with disabilities, evidence on social inequalities in relationships of persons with disabilities is largely missing. The overall objective of this study is therefore to increase our understanding of inequalities in social relationships in persons with disabilities drawing on a sample of persons with spinal cord injury (SCI) from 22 countries. As illustrated in Fig 1, the specific aims of the study are 1) to investigate main effects of individual SES and the countries’ SED on social relationships, and 2) to explore whether social gradients in relationships are moderated by the countries’ SED. We hypothesize that 1a) persons with lower individual SES report poorer relationships; 1b) persons from countries with lower SED report poorer relationships; and 2) social inequalities in relationships are more pronounced in countries with lower SED.

## Materials and methods

### Design

The International SCI community survey (InSCI) was performed in 22 countries between January 2017 and May 2019 covering all continents. The study included 12,330 persons with traumatic or non-traumatic SCI over 18 years of age, who lived in the community and were able to respond to the questionnaire in an available language version [44]. The InSCI questionnaire included 28 items on body functions and structures, 42 items on activities and participation, 26 items on environmental factors, 19 items on personal factors, 2 items on lesion characteristics, and 8 items on appraisal of health and well-being as described in more detail elsewhere [45]. SCI results in complete or partial loss of motor function and sensation below the neurological level of the injury and may severely impact functioning and health and often leads to major disability [46]. Persons with neuro-degenerative disorders, congenital etiologies of SCI, or Guillain Barré syndrome were excluded from the study given that rehabilitation paths and disease developments differ from acquired SCI [44]. National Study Centers were responsible for recruitment, data collection, and the organization of resources to implement the survey. Sampling strategies varied according to local conditions and ranged from random to convenience sampling methods. Depending on local circumstances, countries offered paper-pencil or online questionnaires, telephone or personal interviews, whereby standard operational procedures were applied to guarantee standardization of data collection procedures across countries. Further details regarding methodological features of recruitment and data collection procedures, recruitment results, and sociodemographic and injury characteristics of participants are provided elsewhere [44, 47]. Compliance with national laws and regulatory approvals by Institutional Review Boards or Ethical Committees was mandatory for all countries (S1 Table) and the study conformed to the Helsinki Declaration. Informed consent was sought from each participant in accordance with national regulations.

### Measures

*Social relationships* were operationalized with items on feelings of belongingness, relationship satisfaction, and problems with social interactions. Belongingness was measured with an item from the General Belongingness Scale [48] asking participants whether they feel included when they are with others, rated on a 5-point scale from 0 (not at all) to 4 (completely). Responses were dichotomized into ‘high belongingness’ (3–4) vs. ‘low belongingness’ (0–2). Relationship satisfaction was assessed with a WHOQoL-BREF item [49], assessing the satisfaction with personal relationships on a 5-point scale from 0 (very dissatisfied) to 4 (very satisfied). Responses were dichotomized into ‘very satisfied and satisfied’ (3–4) vs. ‘less than satisfied’ (0–2). Three items from the Model Disability Survey (MDS) measuring problems with social interactions were used asking participants how much of a problem they had with providing care or support for others; with interacting with people; and with intimate relationships [50]. Response options ranged from 0 (extreme problem) to 4 (no problem) and a sum score over the three items ranging from 0–12 was calculated, with higher scores indicating fewer problems with social interactions.

*Individual SES* was operationalized with education, household income, employment status, financial hardship, and subjective social status. Education was assessed in line with the International Standard Classification of Education (ISCED), summing up the total years of formal education, including school and vocational training [51]. Net-equivalent household income in the countries’ currency was calculated by including information on disposable household income, weighted by household composition (number of adults and children) according to OECD criteria [52]. In order to establish comparable categories considering country differences in educational and economic systems, country-based distributional quartiles were built for education and income. Information on employment status was collected with a multiple-choice item on the current employment situation. Persons who indicated working for salary with an employer (also if currently on sick leave) or being self-employed were coded as having paid work and others as having no paid work. Financial hardship was measured with an item on the impact of problematic financial situations on participants life during the past four weeks (not applicable, no influence, made my life a little harder, made my life a lot harder), whereby a 3-categorical variable was used for analysis (not applicable or none, some, massive financial hardship). The MacArthur Scale of Subjective Social Status (SSS) was used to capture the subjective appraisal of one’s position in society, represented by a 10-rung ladder [53]. Participants were asked to cross the rung on which they would place themselves and the variable was used as continuous score ranging from 1–10, with higher values indicating higher SSS.

The *countries’ SED* was operationalized with the Human Development Index (HDI), presenting a statistic composite index of achievements in key dimensions of human development including life expectancy at birth, education, and income ranging from 0–1 [54]. Education was measured as mean years of schooling for adults aged over 25 years and expected years of schooling for children of school entering age. Income was operationalized as gross national income per capita by purchase power parity in USD. The HDI of 2017 was used [54], representing the first year of the InSCI data collection. In order to facilitate the interpretation of effect sizes, the HDI was multiplied with 100 for the main effect models.

#### Potential confounders

The variables gender, age at time of survey, neurological level of injury (paraplegia, tetraplegia), completeness of injury (complete, incomplete), etiology (traumatic, non-traumatic), and time since injury in years were included in model estimation to account for potential confounding due to differential composition of country samples along these characteristics.

### Statistical analysis

Analyses were conducted using STATA version 16.0 for Windows (College Station, TX, USA). First, distributions of variables of interest were described. To address *study aim 1* on *main effects*, social relationship outcomes were regressed on individual SES and countries’ SED using multi-level regressions with a random intercept for country. Logistic regressions were used for the binary outcomes belongingness and relationship satisfaction and linear regression was used for the continuous outcome on social interactions. Odds ratios (OR) for binary outcomes and coefficients for the continuous outcome together with the 95% confidence intervals (CI) and *p*-values are reported. First, we present unadjusted models in which outcomes were separately regressed on each individual SES indicator as well as the countries’ SED (Models 1; 6 models per outcome). Then, models were adjusted for sociodemographic and SCI characteristics (Models 2; 6 models per outcome) and lastly, confounders and all SES indicators were simultaneously included in final models (Models 3, 1 model per outcome). Multi-collinearity between SES indicators was tested by calculating variance inflation factors (VIF), whereby VIFs exceeding 2 indicate the presence of multi-collinearity [55]. VIF values for all SES indicators and confounders were below 1.58, indicating that different indicators are likely to measure distinct SES dimensions. Results shown are based on complete cases. To assess potential bias due to missing values, analyses were repeated with imputed data in sensitivity analysis. Missing values were imputed by multiple imputation (MI), assuming that data were missing at random. MI by chained equations was applied to impute different types of variables [56]. Results from analysis based on imputed data and complete case analysis were compared and no relevant differences between the two strategies were detected (results not shown).

To address *study aim 2* on *moderation*, cross-level interaction terms between individual SES and the countries’ SED were included in regression models 2, estimating the main effects of each individual SES indicator separately together with the countries’ SED indicator and adjusting for sociodemographic and SCI characteristics. The interaction terms provide information on a multiplicative effect between the continuous moderator variable (i.e., the countries’ SED operationalized by the HDI score) and the categorical individual SES variables. In order to allow for differential effects of the individual SES variables on the outcome between countries, a random slope was included for individual SES. Model fit was tested both at the introduction of a random intercept and a random slope using likelihood ratio tests. The likelihood ratio tests showed that the addition of random intercepts and slopes significantly improved the fit in all models. The addition of the random slope was also performed in order to avoid severely anti-conservative statistical inference from testing cross-level interactions without allowing for variance in the association between individual SES and social relationships between countries [33]. The interactions are graphically depicted to illustrate the effects of the countries’ SED on social relationships at different levels of individual SES indicators.

## Results

A description of the study population can be found in Table 1. Around three-quarters of the sample were male and mean age was around 51 years. Incomplete paraplegia was the most common SCI type (34.4%), and complete tetraplegia the least frequent (10.1%). The SCI was caused by trauma in 80.7% of persons and mean time since injury was 13 years. Around 31% of persons had paid work and 17.9% indicated having massive financial hardship. Participants indicated on average 12.1 years of education and a mean of 4.8 on the SSS scale ranging from 1–10. Two-thirds of participants indicated high belongingness and high relationship satisfaction. On average, participants scored 7.7 on the 0–12 social interactions scale, with higher scores indicating fewer problems with interactions. Information on the countries’ SED can be found in the (S2 Table).

**Table 1:** Description of study variables in the 12,330 participants of the InSCI community survey.

Variables [% missing values]	Total
**Categorical variables**	**N (%)**
Male gender [0.3]	8,974 (73.0)
Paid work [0]	3,794 (30.8)
SCI severity [4.0]	
Incomplete paraplegia	4,071 (34.4)
Complete paraplegia	3,335 (28.2)
Incomplete tetraplegia	3,233 (27.3)
Complete tetraplegia	1,196 (10.1)
Traumatic etiology [1.6]	9,797 (80.7)
Education in years [6.5]	
Lowest quartile	2,966 (25.7)
2^nd^ lowest quartile	3,364 (29.1)
2^nd^ highest quartile	2,634 (22.8)
Highest quartile	2,589 (22.4)
Household income [8.1]	
Lowest quartile	2,894 (25.6)
2^nd^ lowest quartile	2,812 (24.8)
2^nd^ highest quartile	2,860 (25.3)
Highest quartile	2,760 (24.4)
Financial hardship [3.8]	
Massive	2,124 (17.9)
Some	3,361 (28.3)
None	6,378 (53.8)
High belongingness [2.8]	7,789 (65.0)
High relationship satisfaction [2.5]	7,883 (65.6)
**Continuous variables**	**Mean (SD); median (IQR)**
Age in years [0.6]	51.2 (15.2); 52 (40–62)
Time since injury in years [2.5]	13.0 (11.8); 9 (4–19)
Education in years [6.0]	12.1 (5.2); 12 (9–15)
Subjective social status (range 1–10) [3.9]	4.8 (2.1); 5 (3–6)
Social interactions (range 0–12) [3.1]	7.7 (3.4); 8 (5–11)

*Abbreviations*: IQR: interquartile range; SCI: spinal cord injury; SD: standard deviation.

### Study aim 1: Main effects of individual SES and countries’ SED on social relationships

Table 2 shows results on the association of individual SES and the countries’ SED with social relationships. All individual SES indicators were consistently associated with social relationships in models 1 and 2. The simultaneous inclusion of all SES indicators in model 3 confirmed associations for employment status, financial hardship and SSS, as persons in paid work, those with higher SSS and those without financial hardship showed a higher likelihood of high belongingness (OR, 95% CI: 1.50, 1.34–1.67; 1.76, 1.53–2.03; 1.16, 1.12–1.19, respectively), high relationship satisfaction (OR, 95% CI: 1.28, 1.15–1.42; 1.97, 1.72–2.27; 1.20, 1.17–1.24, respectively), and fewer problems with social interactions (Coeff, 95% CI: 0.96, 0.82–1.10; 1.93, 1.74–2.12; 0.26, 0.22–0.29, respectively). However, associations with education and income were inconsistent in final models 3.

**Table 2:** Main effects of individual socioeconomic status (SES) and countries’ socioeconomic development (SED) on social relationships: Crude and adjusted results from multi-level models, Odds ratios (OR) or coefficients (Coeff) and 95% confidence intervals (CI).

	High belongingness			High relationship satisfaction			Social interactions		
	1 = high belongingness; 0 = low belongingness			1 = (very) satisfied; 0 = less than satisfied			0–12, higher values = fewer problems with social interactions		
	Model 1	Model 2	Model 3	Model 1	Model 2	Model 3	Model 1	Model 2	Model 3
	OR (95% CI)	OR (95% CI)	OR (95% CI)	OR (95% CI)	OR (95% CI)	OR (95% CI)	Coeff (95% CI)	Coeff (95% CI)	Coeff (95% CI)
***Individual SES***									
**Education (in years)**									
Lowest quartile	Reference	Reference	Reference	Reference	Reference	Reference	Reference	Reference	Reference
2^nd^ lowest quartile	1.14 (1.02–1.27)	1.14 (1.02–1.27)	1.05 (0.93–1.19)	1.18 (1.06–1.32)	1.21 (108–1.35)	1.11 (0.98–1.25)	0.30 (0.14–0.46)	0.18 (0.02–0.34)	0.04 (-0.13–0.20)
2^nd^ highest quartile	1.23 (1.10–1.38)	1.19 (1.06–1.34)	0.95 (0.84–1.09)	1.13 (1.01–1.27)	1.19 (1.06–1.35)	0.96 (0.84–1.09)	0.54 (0.37–0.71)	0.34 (0.17–0.52)	-0.08 (-0.25–0.10)
Highest quartile	1.46 (1.29–1.64)	1.45 (1.28–1.64)	1.01 (0.88–1.16)	1.22 (1.09–1.37)	1.29 (1.14–1.46)	0.89 (0.77–1.02)	0.83 (0.66–1.00)	0.62 (0.45–0.80)	-0.10 (-0.28–0.08)
*p-value*	*<0*.*001*	*<0*.*001*	*0*.*485*	*0*.*003*	*<0*.*001*	*0*.*007*	*<0*.*001*	*<0*.*001*	*0*.*444*
**Household income**									
Lowest quartile	Reference	Reference	Reference	Reference	Reference	Reference	Reference	Reference	Reference
2^nd^ lowest quartile	1.09 (0.98–1.22)	1.06 (0.94–1.19)	0.84 (0.74–0.95)	1.25 (1.12–1.39)	1.22 (1.09–1.37)	0.97 (0.85–1.10)	0.21 (0.04–0.38)	0.18 (0.01–0.35)	-0.33 (-0.50- -0.16)
2^nd^ highest quartile	1.37 (1.23–1.53)	1.33 (1.18–1.49)	0.88 (0.78–1.01)	1.46 (1.30–1.63)	1.45 (1.29–1.63)	0.94 (0.83–1.07)	0.75 (0.59–0.92)	0.75 (0.58–0.92)	-0.16 (-0.33–0.01)
Highest quartile	1.92 (1.71–2.16)	1.86 (1.65–2.10)	0.93 (0.80–1.08)	1.96 (1.75–2.20)	1.95 (1.73–2.19)	0.98 (0.84–1.13)	1.24 (1.08–1.41)	1.19 (1.02–1.36)	-0.31 (-0.50- -0.12)
*p-value*	*<0*.*001*	*<0*.*001*	*0*.*046*	*<0*.*001*	*<0*.*001*	*0*.*827*	*<0*.*001*	*<0*.*001*	*0*.*001*
**Employment status**									
No paid work	Reference	Reference	Reference	Reference	Reference	Reference	Reference	Reference	Reference
Paid work	1.92 (1.76–2.10)	1.93 (1.76–2.13)	1.50 (1.34–1.67)	1.52 (1.39–1.66)	1.64 (1.50–1.81)	1.28 (1.15–1.42)	1.65 (1.52–1.77)	1.41 (1.28–1.54)	0.96 (0.82–1.10)
*p-value*	*<0*.*001*	*<0*.*001*	*<0*.*001*	*<0*.*001*	*<0*.*001*	*<0*.*001*	*<0*.*001*	*<0*.*001*	*<0*.*001*
**Financial hardship**									
Massive	Reference	Reference	Reference	Reference	Reference	Reference	Reference	Reference	Reference
Some	1.31 (1.17–1.48)	1.28 (1.13–1.44)	1.07 (0.94–1.22)	1.62 (1.44–1.82)	1.60 (1.42–1.80)	1.30 (1.14–1.49)	1.20 (1.03–1.37)	1.19 (1.01–1.36)	0.85 (0.67–1.03)
None	2.53 (2.26–2.84)	2.47 (2.19–2.77)	1.76 (1.53–2.03)	2.95 (2.63–3.30)	2.90 (2.58–3.26)	1.97 (1.72–2.27)	2.57 (2.40–2.73)	2.59 (2.42–2.75)	1.93 (1.74–2.12)
*p-value*	*<0*.*001*	*<0*.*001*	*<0*.*001*	*<0*.*001*	*<0*.*001*	*<0*.*001*	*<0*.*001*	*<0*.*001*	*<0*.*001*
**Subjective social status**									
Range 1–10	1.23 (1.21–1.26)	1.23 (1.21–1.26)	1.16 (1.12–1.19)	1.27 (1.25–1.30)	1.27 (1.25–1.30)	1.20 (1.17–1.24)	0.44 (0.41–0.47)	0.43 (0.40–0.46)	0.26 (0.22–0.29)
*p-value*	*<0*.*001*	*<0*.*001*	*<0*.*001*	*<0*.*001*	*<0*.*001*	*<0*.*001*	*<0*.*001*	*<0*.*001*	*<0*.*001*
***Countries’ SED***									
**Human Development Index**									
Range 0–100	1.01 (0.99–1.04)	1.01 (0.99–1.04)	0.99 (0.96–1.02)	1.00 (0.98–1.02)	0.99 (0.97–1.02)	0.97 (0.94–0.99)	0.00 (-0.04–0.05)	0.01 (-0.03–0.60)	-0.04 (-0.09- -0.003)
*p-value*	*0*.*290*	*0*.*340*	*0*.*416*	*0*.*854*	*0*.*491*	*0*.*002*	*0*.*888*	*0*.*563*	*0*.*035*

Model 1: unadjusted. Model 2: adjusted for age, gender, type and completeness of injury, etiology, time since injury. Model 3: Model 2 + all individual-level SES indicators simultaneously entered into models. Results based on complete cases. Abbreviations: SES: socioeconomic status; SED: socioeconomic development.

The countries’ SED was not related to social relationships in unadjusted models. Results from models 3 including all individual SES indicators demonstrate that lower country SED was related to higher relationship satisfaction (OR, 95% CI: 0.97, 0.94–0.99) and less problems with social interactions (Coeff, 95% CI: -0.04, -0.09- -0.003), although effects were small.

### Study aim 2: Cross-level interactions of individual SES and countries’ SED on social relationships

**Fig 2:**
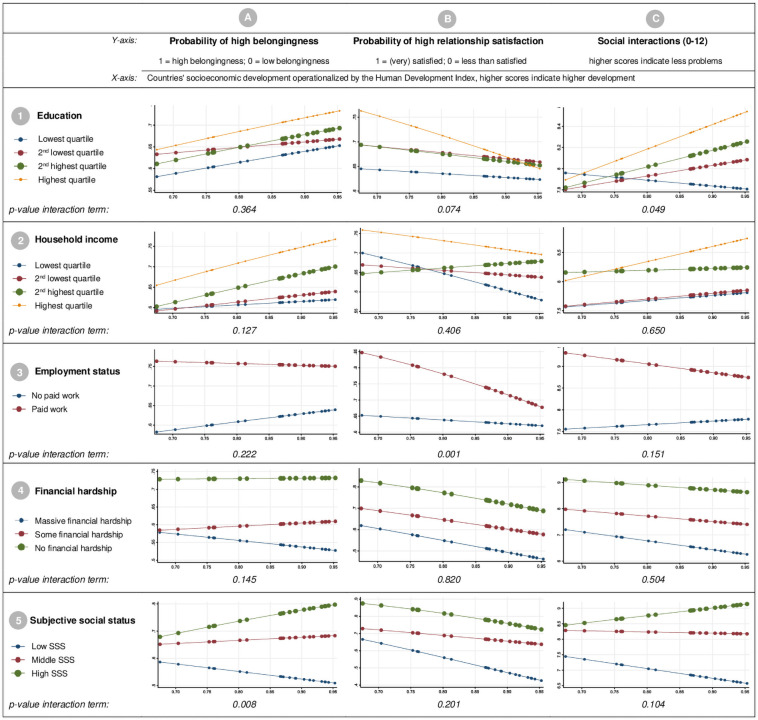
Cross-level interactions between countries’ socioeconomic development operationalized by the Human Development Index and individual SES indicators (1: Education / 2: Household income / 3: Employment status / 4: Financial hardship / 5: Subjective social status) on social relationships (A: Belongingness / B: Relationship satisfaction / C: Social interactions).

Fig 2 illustrates the results of the moderation analysis testing whether associations between individual SES and social relationships vary by the countries’ SED. Graphs 1A and 1B for education indicate that educational inequalities in belongingness and relationship satisfaction are relatively stable across countries, while graph 1C shows that educational inequalities in social interactions were larger in higher SED countries (*p-value* interaction term *0*.*049*). Graphs for household income indicate fairly stable income gradients for belongingness across countries (graph 2A), but show that income inequalities were inconsistent for relationship satisfaction and social interactions, whereby trends for income gradients were only observable in higher income countries (*p-values* interaction terms *>0*.*05*).

Graphs 3A-C for employment show a clear trend towards larger inequalities in social relationships between persons in paid work and unemployed persons in lower SED countries, whereby moderation effects were most pronounced for relationship satisfaction (graph 3B, *p-value* interaction term *0*.*001*). Results for financial hardship provide evidence for a stable social gradient in social relationships across all countries (graphs 4A-C), with a slight trend for larger inequalities in belongingness in higher SED countries (graph 4A, *p-value* interaction term *0*.*145*). Graphs 5A-C for SSS indicate a consistent trend towards higher inequalities in social relationships according to the SSS in higher SED countries, whereby differences were strongest for belongingness (graph 5A, *p-value* interaction term *0*.*008*), followed by problems in social interactions (graph 5C, *p-value* interaction term *0*.*104*).

## Discussion

This study provides initial evidence for social inequalities in relationships of persons with SCI across different geographical settings considering socioeconomic inequalities at both, individual and country-level. Our findings mainly support the hypothesis that lower individual SES is associated with poorer social relationships as we observed that paid work, absence of financial hardship and higher SSS were consistently related to higher feelings of belongingness, higher relationship satisfaction, and fewer problems with social interactions. However, support for this hypothesis was weaker for the classical SES indicators education and income when all SES indicators were tested simultaneously. The hypothesis that people from countries with higher SED generally report better social relationships was not confirmed in our analysis as even the opposite was found for relationship satisfaction and social interactions. Finally, the hypothesis stating that social inequalities in relationships were more pronounced in countries with lower SED received only partial empirical support as some interactions pointed in the opposite direction, showing larger inequalities in higher SED countries.

### Main effects

Results for people with SCI presented in this study are mostly in line with previous general population findings demonstrating that persons with lower SES are disadvantaged in their social relationships [6, 10–17, 57]. Also, one of the few available studies in persons experiencing disability documented that financial hardship was associated with lower relationship satisfaction [38]. Comparison of our results with previous findings is however limited as earlier studies mainly investigated social networks and perceived or received social support. Moreover, studied SES indicators were mostly restricted to education, income and occupational position and only few studies included more subjective aspects of SES, such as perceived financial hardship or SSS.

Our results maintain the notion that education and income have a reduced predictive value for social relationships in comparison to more subjective SES indicators and employment status when they are investigated simultaneously. A possible explanation could be that more subjective indicators mediate the effect of income and education on relationships, thus mitigating effects of the latter in models considering both types of indicators. This interpretation is supported by previous studies showing that SSS was more strongly linked to health than the traditional SES indicators education and income. Individual perception of one’s place in the social hierarchy is possibly more closely related to negative affect states and emotions than objective indicators [58, 59], providing also an argument for their stronger impact on social relationships as compared to objective indicators. Moreover, financial hardship might more adequately captures poverty than the indicator income, and poverty obviously affects social relationships through limited social participation [60, 61], possibly due to the restricted financial resources to participate in leisure activities (e.g., specialized sports equipment, club memberships, transportation to events), or to invite or visit others to foster friendships and it is likely that poor social participation in lower SES groups affects relationship satisfaction and hampers feelings of belongingness. Unemployment further restricts social interactions as work-related social networking opportunities are absent and the risk of social exclusion increases due to stigma [62]. Moreover, persons with lower SES are prone to experience higher psychosocial distress [63, 64], potentially enhancing dysfunctional social interactions. A study supporting this assumption observed for example that financial hardship reduced the relationship satisfaction in couples, possibly through deteriorated dyadic coping and increased negative behaviors, leading to distress in the partner relationships [65].

Our data did not support the hypothesis that people from countries with higher SED report better social relationships. Although effect sizes were small, the countries’ SED was negatively related to relationship satisfaction and social interactions when individual SES was adjusted for. One potential explanation is that formal help is less available in countries with lower SED and that informal care from family, friends and within neighborhoods is comparably higher [66], leading to higher relationship satisfaction. Earlier findings from the InSCI study also showed that persons from higher SED countries more often live alone [67]. This may not only affect their relationship satisfaction as everyday social interaction with persons in the same household is missing, but could also affect their social participation outside the home as household members could be instrumental in overcoming environmental barriers.

### Moderation

Results of the moderation analysis suggest that socioeconomic gradients in social relationships partly vary according to the countries’ SED. Such variation was observed for the extent of social inequalities in relationships according to the countries’ SED for individual SES indicators education, employment status, and SSS. The finding that paid work was more important for relationship satisfaction in lower SED countries could be explained by the fact that unemployed persons have restricted possibilities to connect with people outside the home because platforms to meet people (e.g., sports clubs, associations for persons with disabilities) are possibly less available and/or less accessible for persons with physical disabilities in lower SED countries. Although empirical findings supporting this assumption are missing, it is likely that having a disability and being unemployment in low SED settings is related to double-stigmatization and paid work therefore has a larger impact on opportunities to form and maintain functional relationships. The finding that inequalities in social relationships were in tendency larger according to SSS in higher SED countries might point at differences in the emotional reactions related to perceiving own social status as low. In higher SED countries, population average of education is higher and the prevalence of persons living below the poverty line is lower. People feeling marginalized in the social hierarchy might thus show stronger emotional reactions than in countries with lower SES on the population level. The perception of low social status and related enhanced negative emotional reactions in higher SED countries might lead to increased feelings of social exclusion and poorer satisfaction with social interactions. However, empirical testing of such assumptions is yet to be undertaken.

### Limitations

The generalizability of results to the total population of individuals with SCI in participating countries might be limited due to sampling bias as 14 of 22 countries relied on convenience sampling and only eight countries applied random sampling strategies. Therefore, this study does not intend to provide representative data on social relationship constructs, but intends to gain insights into associations between socioeconomic conditions on the individual and country-level and social relationships in persons experiencing a physical disability. As no information on social relationships or SES is available for non-responders, we cannot evaluate whether survey participation was independent of these factors. We further cannot assess whether self-report of indicators such as education and income has led to biased responses, as for example information on income might be prone to social desirability bias. Also, this study only investigated three specific aspects of social relationships assessed with five items, and we cannot conclude that our findings are representative for the full spectrum of functional aspects of social relationships. Finally, reverse causation cannot be excluded for some constructs, as for example persons with many problems with social interactions might face difficulties in maintaining paid work, rate their social status as low, or have limited access to pursue higher education.

## Conclusions

This study provides evidence for social inequalities in functional aspects of social relationships. Paid work, financial hardship and SSS were particularly important for social relationships in the analyzed sample of people experiencing physical disability, namely SCI. Social inequalities in relationships were partly moderated by the countries’ SED with paid work playing a greater role in countries with lower SED and SSS being more important in countries with higher SED. As social relationships provide important health resources, rehabilitation services supporting persons in establishing and maintaining social relationships should specifically target persons from lower SES groups. Moreover, policies and occupational rehabilitation services to increase labour market participation of persons with SCI are crucial as paid work not only provides valuable resources for social relationships but also helps preventing financial hardship, which also plays an important role for social relationships.

## Supporting information

S1 Table Ethics committees or review boards approvals in the 22 InSCI countries. (DOCX)

S2 Table The countries’ socioeconomic development measured by the Human Development Index (HDI) as composite index of life expectancy at birth, years of education, and gross national income per capita by purchase power parity in USD for participating countries: Original score (0–1) as of 2017, with higher scores indicating higher socioeconomic development. (DOCX)
