# Supplementary material for: Socioeconomic status and social relationships in persons with spinal cord injury from 22 countries: Does the countries’ socioeconomic development moderate associations?
Source: PLoS One. 2021 Aug 13;16(8):e0255448. doi: 10.1371/journal.pone.0255448 (PMC8362947; doi:10.1371/journal.pone.0255448)
Supplement: S1 Table — (DOCX) [file pone.0255448.s001.docx]

**S1 Table. Ethics committees or review boards approvals in the 22 InSCI countries**

| **Country** | **Name of ethics committee or institutional board responsible for ethics approval of the InSCI study** | **Approval number** | **Approval date** | **Form of consent** |
| --- | --- | --- | --- | --- |
| Australia | Northern Sydney Local Health District Human Research Ethics Committee;  Australian Institute of Health and Welfare Ethics Committee | HREC/16/HAWKE/495; EO2017/1/341 | June 7 2017; Jan 4 2018 | D |
| Brazil | Comissão de Ética para Análise de Projetos de Pesquisa do Hospital das Clínicas da Faculdade de Medicina da Universidade de São Paulo, CAPPesq | 97049118.8.0000.0068 | Nov 8 2018 | A |
| China | Ethics committees of the first affiliated hospital of Nanjing Medical University, Nanjing, Jiangsu Province; Ethics Committee of Sichuan University, Sichuan Province, Chengdu | No. 2018-SR-004 (Jiangsu)  No. K2017053 (Sichuan) | March 7 2018;  Jan 22 2018 | B, C |
| France | Comité de Protection des Personnes | Ref : 180304 | April 10 2018 | A |
| Germany | Ethic Committee of Hannover Medical School | 7374 | Feb 13 2017 | B |
| Greece | Scientific/Ethical Committee of General Hospital 'G. Gennimatas' Athens | 20257/1.8.2016 | Aug 1 2016 | A, B |
| Indonesia | Health Research Ethics Committee, National Institute of Health Research and Development | LB.02.01/2/KE.342/2017 | 15 Nov 2017 | A |
| Italy | Comitato Etico Interaziendale AOU 'Maggiore della Carità' di Novara, ASL BI, ASL NO, ASL VCO | ItaSCI, 1, 25-01-2018 | April 27 2018 | A |
| Japan | Research Ethics Committee of Wakayama Medical University | 2079 | July 12 2017 | A |
| Lithuania | Vilnius Regional Committee for the Ethics of Biomedical Research | 158200-17-907-421 | Mai 9 2017 | A, B |
| Malaysia | Medical Research and Ethics Committee, Ministry of Health | NMRR-16-2747-28885(IIR) | July 14 2017 | A |
| Morocco | Hospital and University Ethics Committee of Fez | 03/17 | July 20 2017 | A |
| Netherlands | Medical Ethics Board University Medical Center Utrecht | WAG/mb/17/024763 | Aug 16 2017 | A |
| Norway | Regional Committee for Medical and Health Research Ethics, South East | 2016/1184/REK sør-øst | Sept 21 2016 | A, B |
| Poland | Bioethical Committee of the Medical University of Lodz | RNN/198/16/KE | July 12 2016 | A, B |
| Romania | Ethical Committee of Rehabilitation Hospital Felix Spa | 2228/06.03.2017 | March 3 2017 | A, B |
| South Korea | Institutional Review Board of National Rehabilitation Center | NRC-2016-05-039 | Nov 9 2016 | A, B |
| South Africa | Biomedical Science Research Ethics Committee of the University of the Western Cape | BM/16/3/24 | Oct 24 2016 | A, C (recorded) |
| Spain | Ethical Committee of Hospital Universitari Vall d’Hebron, Hospital Universitario de Cruces, Hospital Universitario Materno Infantile de Gran Canaria, Hospital Universitario Virgen del Rocio | PR(ATR)285/2016 | Oct 2016 | A |
| Switzerland | Ethical Committee of Northern and Central Switzerland | 11042 PB_2016-02608 | Dec 21 2016 | A, B |
| Thailand | Ethical Committee of Faculty Medicine, Chiang Mai University | REH-2559-04167 | Dec 9 2016 | A |
| United States | University of Vermont Institutional Review Board | CHRBSS:16-574 | Nov 21 2017 | A, E |
| Note: A=written consent; B=questionnaire completion considered as implicit consent; C=oral consent; D=waiver of consent; E=electronic consent | | | | |
