# Supplementary material for: Socioeconomic status and social relationships in persons with spinal cord injury from 22 countries: Does the countries’ socioeconomic development moderate associations?
Source: PLoS One. 2021 Aug 13;16(8):e0255448. doi: 10.1371/journal.pone.0255448 (PMC8362947; doi:10.1371/journal.pone.0255448)
Supplement: S2 Table — (DOCX) [file pone.0255448.s002.docx]

**S2 Table.** **The countries' socioeconomic development by the Human Development Index as composite index of life expectancy at birth, years of education, and gross national income per capita by purchase power parity in USD for participating countries: Original score (0-1) as of 2017, with higher scores indicating higher socioeconomic development.**

| **Country** | **Human Development Index 2017** |
| --- | --- |
| Norway | 0.953 |
| Switzerland | 0.943 |
| Germany | 0.938 |
| Australia | 0.937 |
| Netherlands | 0.932 |
| United States | 0.919 |
| Japan | 0.913 |
| South Korea | 0.904 |
| Spain | 0.891 |
| France | 0.890 |
| Italy | 0.881 |
| Greece | 0.871 |
| Poland | 0.868 |
| Lithuania | 0.866 |
| Romania | 0.813 |
| Malaysia | 0.802 |
| Thailand | 0.762 |
| Brazil | 0.760 |
| China | 0.753 |
| Indonesia | 0.704 |
| South Africa | 0.704 |
| Morocco | 0.675 |
